# Compliance and Knowledge of Healthcare Workers Regarding Hand Hygiene and Use of Disinfectants: A Study Based in Karachi

**DOI:** 10.7759/cureus.7036

**Published:** 2020-02-18

**Authors:** Jawad Ahmed, Farheen Malik, Zahid Ali Memon, Taha Bin Arif, Aiman Ali, Sundus Nasim, Junaid Ahmad, Muhammad A Khan

**Affiliations:** 1 Internal Medicine, Dow University of Health Sciences, Karachi, PAK; 2 Pediatrics, Dow University of Health Sciences, Karachi, PAK; 3 Surgery, Dow University of Health Sciences, Karachi, PAK; 4 Surgery, Civil Hospital, Karachi, PAK; 5 Internal Medicine, Liaquat University of Medical and Health Sciences, Jamshoro, PAK

**Keywords:** hand hygiene, nosocomial infections, hand disinfectants, awareness, patient safety, health care associated infections

## Abstract

Background

Hand hygiene is the cardinal step in combating various healthcare-associated infections. These infections are a cause of 37,000 deaths in Europe and 100,000 deaths in the United States annually. Thus, prevention of their spread is of utmost importance today. A study conducted in a tertiary care center in Karachi found that 17% of the medical professionals were aware of the World Health Organization (WHO) guidelines on hand hygiene while only 4.9% followed these hand-washing techniques. Lack of hand hygiene practice and awareness has raised a need to reassess infection control in hospitals. There is currently undisputed proof that adherence to hand cleanliness diminishes the danger of transmission of various infections.

Methods

A questionnaire-based cross-sectional study was conducted at Dr. Ruth K.M. Pfau Civil Hospital, Karachi in January 2019. Data from 212 participants who met the inclusion criteria were analyzed. A three-part questionnaire was used for the hospital staff who had been present at the hospital for at least six hours and had attended to the patients during the last three continuous working days. Staff members who visited the hospital but did not attend to any patients or those who had been present at the hospital for less than six hours were excluded. Collected data were analyzed using Statistical Package for Social Science (SPSS) version 23.0 (IBM, Armonk, NY).

Results

A total of 212 individuals (74 doctors, 66 nurses, 52 technicians, and 20 ward assistants) agreed to participate in our study, of which 124 were females. The compliance with hand disinfectant use before and after every patient contact was found to be 12.3%. The use of disinfectant was found to be more among males than females (mean 7.88 times for males vs. 6.20 for females) and the younger individuals were more compliant with hand hygiene practices; 62.73% of participants were aware of the WHO guidelines regarding hand hygiene and 65.56% were aware of hospital-acquired infections. However, nearly half of the participants (45.75%) had never attended a formal lecture on the subject and more than half (62.26%) of the participants were unenlightened about the complications of hospital-acquired infections.

Conclusions

Hand hygiene is a basic requirement for every medic and paramedic in a hospital setting today. Keeping in mind the drastic consequences of the spread of hospital-associated infections, it is evident that hand hygiene should be stressed upon. The rising incidence of nosocomial infections and their complications can be prevented by raising awareness about hand hygiene practices. There is a need to further investigate the application of and adherence to the basic guidelines on hand hygiene. Our results indicate that this issue should be tackled through a multidimensional approach.

## Introduction

Healthcare-associated infections are a major obstacle to achieving pinnacle healthcare. With a soaring number of 37,000 deaths from 4,544,100 infections in the European Union annually, and about 2,000,000 infections and 100,000 deaths annually in the United States, these infections pose a serious threat to millions of people worldwide [1]. An integral method of prevention of the spread of nosocomial infections lies in our own hands. Hand Hygiene is a simple and cost-effective method that plays a vital role in controlling the outbreak of infections. Lack of proper hand hygiene practices acts as a source of the spread of common healthcare-associated infections that may affect the urinary, respiratory, and gastrointestinal tract, as well as surgical sites [2]. The significance of hand hygiene was brought to light again in 2002 through the revised guidelines published by the Centres for Disease Control and Prevention (CDC), which recommended the use of the alcohol-based solution for invisible hand decontamination and usage of soap and water for visible contamination [3]. In a study by Girou et al., alcohol-based hand rubs were found to be significantly more effective than washing hands with soap in reducing bacterial contamination [4].

The compliance of hand hygiene among healthcare workers, unfortunately, has been mediocre. According to the Society for Healthcare Epidemiology of America, only 31% of the healthcare providers were well informed about proper hand hygiene practices. Apart from healthcare workers, medical students are also involved significantly in patient care. One might assume that medical students are aware of and compliant with these sanitation practices, yet, a study conducted during Observed Structural Clinical Examinations (OSCE) in Saudi Arabia found that hand hygiene compliance among medical students was only 17% [5]. Factors leading to these unsatisfactory results include lack of knowledge and awareness, high-stress work environment, misconceptions about hand hygiene, and poor practices by peers and mentors [6].

Pakistan is among the countries where infectious diseases are identified as a major threat and a leading cause of patient morbidity and mortality. A study conducted among the doctors, nurses, and medical students of Allied Hospitals of Rawalpindi Medical University revealed that, even though the medical students were well informed about hand hygiene, only 37% of healthcare professionals practiced hand washing, whereas the WHO technique of hand washing was followed by only 19% of this 37% [7]. Another study by Anwar MM et al. found that among 211 physicians of a tertiary care hospital of Karachi, only 4.9% of the respondents practiced proper hand hygiene and only 17% were well informed about the WHO guidelines on hand hygiene [8]. 

Despite the high prevalence of infections in Pakistan, data regarding hand hygiene among healthcare workers is limited and mostly outdated. Thus, we sought to conduct a study with the primary aim of finding the frequency of utilization of alcohol disinfectant by hospital staff in a tertiary care hospital in Karachi, Pakistan. The secondary aim of this study was to assess the knowledge of hospital staff regarding various aspects of hand hygiene.

## Materials and methods

Study duration and population

A questionnaire-based cross-sectional study was carried out for a period of one month at Ruth K.M. Pfau Civil Hospital, a government-run tertiary care center based in Karachi in January 2019. The population under study consisted of hospital staff members including doctors, nurses, technical staff, and ward assistants.

Inclusion and exclusion criteria

All the hospital staff members who had been present in the hospital for a minimum of six hours and had attended to patients during the last three continuous working days were included. All the staff members who had worked less than six hours on any day in past three days were excluded and so were the staff who were in the hospital but did not attend to patients. Based on these exclusion criteria, data from eight participants were excluded from the analysis. 

Sample size and study design

We approached 304 staff members and 220 agreed to take part in our study. The cooperation rate was 72.36%. The sample size was calculated (through OpenEpi.com) to be 207, with a 95% confidence interval (CI) and a 5% error margin. Using convenience sampling, 212 participants were included in the study after receiving informed consent. Any questions that the participants had regarding the study were addressed in detail. All participants were informed that their responses would remain confidential and that they had the right to withdraw from the study as per their wish. The questionnaire consisted of three parts: (1) demographic profile, (2) information regarding duty hours and hand disinfectant use during these hours, and (3) knowledge regarding the importance of hand disinfectant use in the prevention of various hospital-acquired infections.

Statistical analysis

All the data were entered into Statistical Package for Social Science (SPSS) software version 23.0 (IBM, Armonk, NY) for analysis. Results are presented as means with standard deviations (for continuous variables) and percentages and frequencies (for categorical variables). Chi-square and independent-sample t-test were used to analyze relationships between different variables and a value of p: <0.05 was considered statistically significant in all cases.

## Results

A total of 220 participants agreed to take part in our study, of which 212 were included after applying the exclusion criteria. Among these, 88 (41.5%) were males and 124 were (58.5%) females. More than half of the staff (n = 114; 53.8%) were from the internal medicine department, followed by 80 (37.7%) from the surgical, and 18 (8.5%) from the dental department. The majority of the participants were doctors (34.90%), followed by nurses (31.13%), technical staff (24.53%), and female ward assistants (9.43%). Most of the staff worked 6-8 hours each day. The mean age of staff members was 30.82 ±8.69 years. The demographic profile of the participants is summarized in Table 1.

**Table 1:** Demographic profile of the study participants

Basic characteristics	Variables	n (%)
Gender	Male	88 (41.5)
	Female	124 (58.5)
Age groups	20–40 years	182 (85.8)
	41–60 years	30 (14.2)
Departments	Medicine	114 (53.8)
	Surgery	80 (37.7)
	Dental	18 (8.5)
Workgroups	Doctors	74 (34.9)
	Nurses	66 (31.13)
	Technicians	52 (24.53)
	Ward assistants (Ayas)	20 (9.43)

Use of hand disinfectant

Men on average used disinfectant significantly more frequently than females (mean 7.88 for males vs. 6.20 for females; p: <0.001). Among doctors, males on average used hand disinfectants more frequently than females; however, the reverse was true in the rest of workgroups. A significant difference was seen in the use of hand disinfectant between both age groups (p: 0.008). The younger age group of 20-40 years had a higher frequency of hand disinfectant use than the elder age group of 41-60 years (Table 2).

**Table 2:** Comparison of use of hand disinfectant between different age groups in males and females

Gender	Age group	Mean hand disinfectant use on day 1	Mean hand disinfectant use on day 2	Mean hand disinfectant use on day 3
Male	20–40 years	7.74	7.95	8.29
	41–60 years	7.17	7.17	7.17
Female	20–40 years	6.30	6.49	6.64
	41–60 years	4.33	4.67	4.67

**Figure 1:**
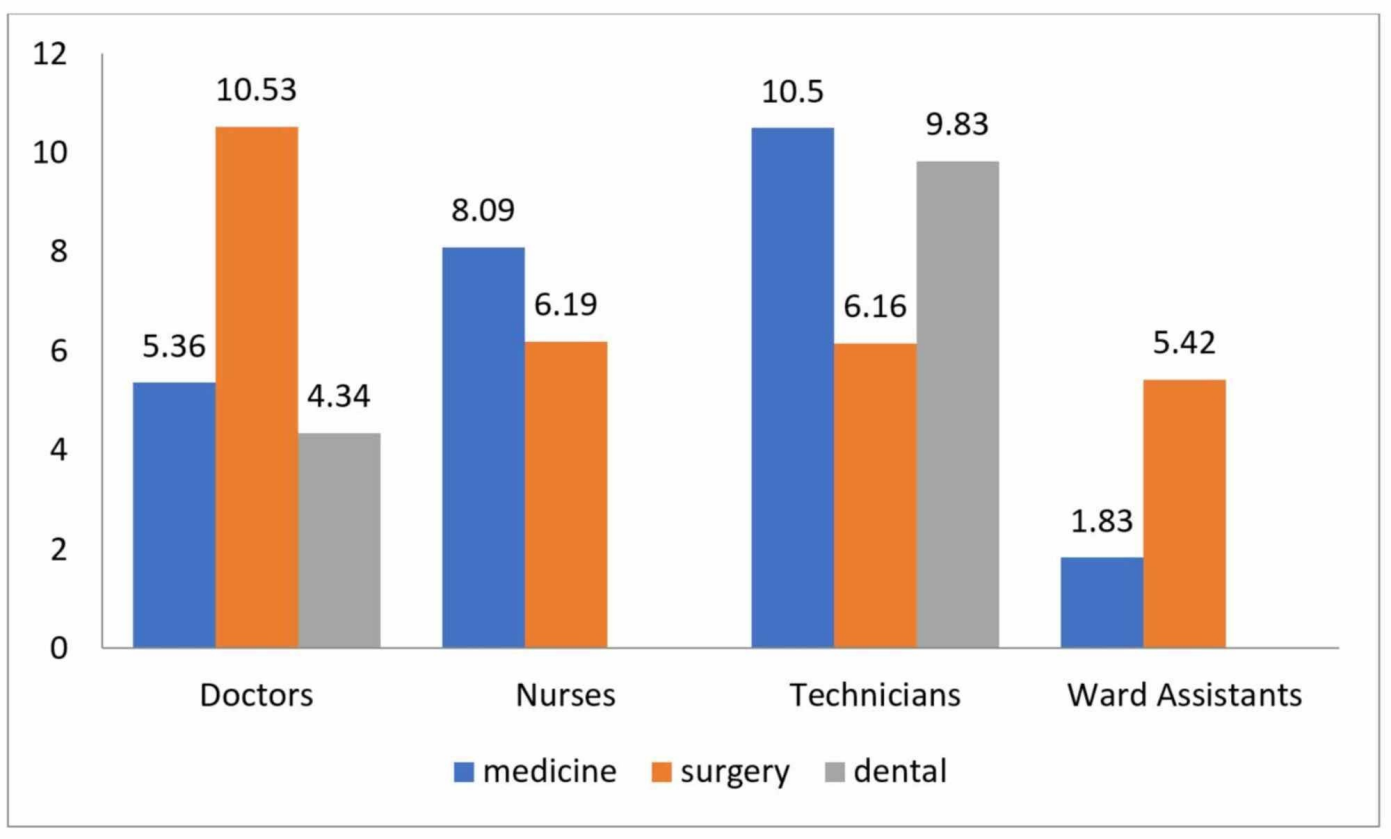
Average hand disinfectant use (department- and workgroup-wise distribution) The values on top of bar graphs represent mean usage

The proportion of the staff that consistently used hand disinfectant before and after attending to every patient was 12.3%. Most frequent disinfectant use over the course of three days was in the surgery department, followed by medicine, and then the dental department (7.19, 6.90, and 5.5 times respectively). Doctors and ward assistants of surgery department used disinfectant more frequently than those of the medicine department, but the nurses and technical staff of the medicine department reported a higher rate of use of disinfectant than those in the surgical department (Figure 1).

Lack of time and lack of disinfectant in close proximity were identified as the most common reasons for not using hand disinfectant (43% and 27.4% respectively). The responses of 186 participants who denied using hand disinfectant before and after every patient contact are given in Table 3.

**Table 3:** Reasons for inadequate use of hand disinfectants (N = 186)

Reason for not using hand disinfectant	Number of participants, n (%)
Lack of time	75 (40.3)
Lack of disinfectant in close proximity	51 (27.4)
Patient overload makes it impossible	26 (14)
Using it after every patient is not important	31 (16.7)
No role in preventing disease spread	3 (1.6)

Knowledge about hand hygiene and its importance

A series of questions were asked to gain insight into awareness of different aspects related to hand hygiene. When inquired about WHO guidelines regarding hand hygiene and the use of disinfectant, more than half (62.73%) of the participants reported being aware of them. Almost half of the participants (97; 45.75%) had never attended or received formal workshops/lectures regarding the importance of hand hygiene. Significantly, 65.56% of the participants had adequate awareness regarding ‘nosocomial infections’ and ‘hospital-acquired infections.’

**Figure 2:**
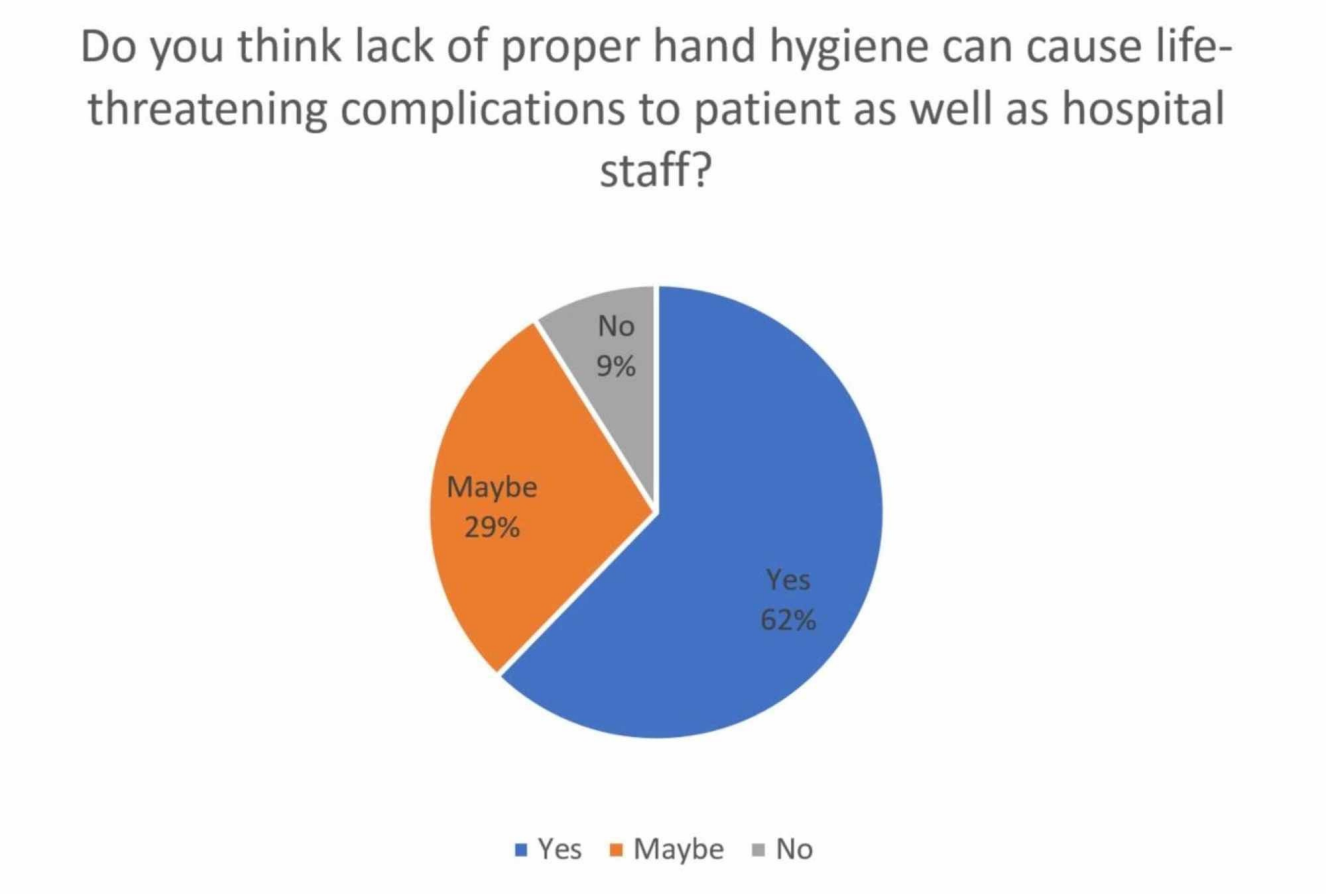
Awareness among participants about lack of hand hygiene and its consequences

An overwhelming 44.81% of the hospital staff (45 nurses, 30 technical staff, and 20 ward assistants) responded to being unaware of the complications of nosocomial infections. Nearly two-third (62.26%) of the participants were aware that the lack of proper hand hygiene could cause life-threatening complications to the patient as well as hospital staff (Figure 2). It was observed that 134 (63.20%) participants were conscious of immune-compromised patients in their wards and 55 (41.04%) said they ensured to take special care and precaution while handling them.

## Discussion

Studies have shown that improved compliance with hand hygiene led to a notable reduction in infection rates[9]. However, keeping up optimal hand hygiene poses a hurdle in most healthcare settings. Lack of adequate knowledge of guidelines, long working hours, empty hand hygiene products, understaffing or overcrowding, skepticism regarding the value of hand hygiene, and a belief that glove use obviates the need for hand hygiene are some of the factors responsible for poor compliance [10]. 

The present study demonstrated that overall hand hygiene compliance was 12.3% among the study participants. This finding was found to be higher than in studies conducted at Wachemo University Teaching Hospital, Ethiopia (9.2%) and Africa Ghana Teaching Hospital (12%) [11,12]. In contrast, studies conducted in Kuwait (33.4%), India (43.4%), and North West Nigeria (55.2%) have shown considerably higher compliance rates than our study [2,13,14]. This variation might be due to a higher prevalence of inadequate knowledge of nosocomial infections among healthcare providers, and inaccessibility of hand hygiene products and facilities in our setting. Despite a greater ratio of females in our study, we found that the usage of hand disinfectant was approximately twice among males. It must be noted, however, that the gender difference in hand hygiene measures seen in our study population was seen mainly among doctors. In other working groups, females were consistently found to have a higher rate of disinfectant use. This is in line with a study by White et al, which demonstrated that female healthcare providers wash hands more than males [15].

This study also demonstrated that young individuals were more likely to follow hand hygiene protocol. The immense rise in hospital-acquired infections in recent years has put more emphasis on preventive measures. It has been observed that interactive educational programs combined with a free supply of resources have significantly raised compliance [16]. Factors affecting compliance can be related to healthcare staff, their clinical life, and the environment they are exposed to [17]. Lack of time and unavailability of disinfectant nearby were the dominant reasons documented by the individuals in this study. Due to overcrowding and long working hours in our setting, it is possible that the staff does not have adequate time to use alcohol-based hand rubs after every patient contact. In addition, the role of government in ensuring the availability of resources plays a primary role in influencing compliance. A low-middle income country with inadequate resources faces great difficulty to meet international standards, which could explain the reason for reduced compliance. ​

The department of surgery was found to be more compliant with hand hygiene in this study. This is likely due to the special emphasis put on maintaining adequate hand hygiene in the operation theatres. In the medicine department, technicians and nurses, not doctors, were found to be more in contact with patients; hence they are likely to use disinfectant more frequently, as seen in our study. This finding is supported by a study by Randle et al. [18]. 

The second part of this study investigated the knowledge of nosocomial infections and hand hygiene guidelines by WHO among the participants. In 2019 the WHO introduced ‘Clean care for all- it’s in your hands’ mission of universal health coverage, which focuses on the urgent need for access to healthcare for all people worldwide [19]. In our study, approximately two-thirds of the population reported being aware of nosocomial infections, and more than half of the participants knew about WHO hand hygiene guidelines. A study conducted in Faisalabad, Pakistan found that while the majority of the nursing staff had adequate knowledge, their practices were not in line with their knowledge scores [20]. Similarly, when inquired about nosocomial infections and its prevention among the nursing staff of a tertiary care hospital in Rawalpindi, a significant gap between knowledge and practice was observed. Education-based workshops and seminars were the major sources of awareness while a few mentioned websites as their source [21]. Results from our study are in harmony with those from previous studies. A vast majority of the participants were aware of the complications related to poor hand hygiene compliance, especially in immunocompromised patients. However, compliance with hand hygiene was still limited. Training can have a positive impact on the improvement of practices. Besides, the use of effective methods of disinfection, continuous timely training, and knowledge improvement can reduce the frequency of hospital-acquired infections [22]. In a tertiary healthcare setting where multiple patients are treated at the same time, people are highly susceptible to pathogens and can easily catch infections. ​Hence, a proper surveillance system with innovative educational training programs that can motivate healthcare workers should be introduced. ​

There are some limitations to this study. First, our sample size was small and covered only one hospital in Karachi. A multi-centered study giving an overview of the practices of the staff of different hospitals can provide better results. Secondly, the scope of this study was limited to inquiring about knowledge and compliance with the usage of hand disinfectants. Such interview-based studies can be susceptible to Hawthorne bias. A wider, more focussed study should be conducted to examine the level of adherence to the WHO guidelines and their implementation in all hospitals of Pakistan.

## Conclusions

Our study population had adequate knowledge about nosocomial infections and their relationship with hand hygiene compliance. However, a significant gap between the knowledge and practices of participants was observed. Male healthcare providers used disinfectant more frequently than women altogether, especially in the surgical department. A lack of adherence to guidelines was noticed among nurses, ward assistants, and technicians. This study highlights the need for conducting training on infection control and prevention in a healthcare setting, which would stress on maintaining adequate hand hygiene through disinfectants with strict supervision.

## References

[1] Gesser-Edelsburg A, Cohen R, Zemach M, Halavi AM (2020). Discourse on hygiene between hospitalized patients and health care workers as an accepted norm: making it legitimate to remind health care workers about hand hygiene. Am J Infect Control.

[2] Al-Wazzan B, Salmeen Y, Al-Amiri E, Abul A, Bouhaimed M, Al-Taiar A (2011). Hand hygiene practices among nursing staff in public secondary care hospitals in Kuwait: self-report and direct observation. Med Princ Pract.

[3] Boyce JM, Pittet D (2002). Guideline for hand hygiene in health-care settings. Recommendations of the Healthcare Infection Control Practices Advisory Committee and the HICPAC/SHEA/APIC/IDSA Hand Hygiene Task Force. Society for Healthcare Epidemiology of America/Association for Professionals in Infection Control/Infectious Diseases Society of America. MMWR Recomm Rep.

[4] Girou E, Loyeau S, Legrand P, Oppein F, Brun-Buisson C (2002). Efficacy of handrubbing with alcohol based solution versus standard handwashing with antiseptic soap: randomised clinical trial. BMJ.

[5] Qasmi SA, Mahmood Shah SM, Wakil HYI, Pirzada S (2018). Guiding hand hygiene interventions among future healthcare workers: implications of knowledge, attitudes, and social influences. Am J Infect Control.

[6] Erasmus V, Daha TJ, Brug H, Richardus JH, Behrendt MD, Vos MC, van Beeck EF (2010). Systematic review of studies on compliance with hand hygiene guidelines in hospital care. Infect Control Hosp Epidemiol.

[7] Munir M, Maqbool M, Bilal S, Hussain M, Ghani Z, Yaqub A (2018). Handwashing practices in health care professionals of allied hospitals of Rawalpindi medical university. Ann Pak Inst Med Sci.

[8] Anwar MA, Rabbi S, Masroor M, Majeed F, Andrades M, Baqi S (2009). Self-reported practices of hand hygiene among the trainees of a teaching hospital in a resource limited country. J Pak Med Assoc.

[9] Hugonnet S, Perneger TV, Pittet D (2002). Alcohol-based handrub improves compliance with hand hygiene in intensive care units. Arch Intern Med.

[10] Al Ghafari Z, AbuRuz ME (2019). Hand hygiene knowledge, attitude and barriers among Jordanian nurses. Int Med J.

[11] Meshesha AA, Tiruneh YA, Asegid A, Ayele Y (2017). Hand hygiene compliance and associated factors among health professionals in Wachemo university hospital, Hossaena, South West Ethiopia. IJIRD.

[12] Owusu-Ofori A, Jennings R, Burgess J, Prasad PA, Acheampong F, Coffin SE (2010). Assessing hand hygiene resources and practices at a large African teaching hospital. Infect Control Hosp Epidemiol.

[13] Sharma S, Sharma S, Puri S, Whig J (2011). Hand hygiene compliance in the intensive care units of a tertiary care hospital. Indian J Community Med.

[14] Garba MB, Uche LB (2019). Knowledge, attitude, and practice of hand washing among healthcare workers in a tertiary health facility in northwest Nigeria. J Med Trop.

[15] White C, Kolble R, Carlson R, Lipson N (2005). The impact of a health campaign on hand hygiene and upper respiratory illness among college students living in residence halls. J Am Coll Health.

[16] Sjöberg M, Eriksson M (2020). Hand disinfectant practice: the impact of an education intervention. Open Nurs J.

[17] Mathur P (2011). Hand hygiene: back to the basics of infection control. Indian J Med Res.

[18] Randle J, Clarke M, Storr J (2006). Hand hygiene compliance in healthcare workers. J Hosp Infect.

[19] Peters A, Borzykowski T, Tartari E, Kilpatrick C, Mai HCS, Allegranzi B, Pittet D (2020). "Clean care for all-it’s in your hands”: the May 5th, 2019 World Health Organization SAVE LIVES: Clean Your Hands campaign. J Infect Dis.

[20] Sadaf S, Inayat S, Afzal M, Hussain M (2018). Nurse’s knowledge and practice regarding prevention of surgical site infection at Allied Hospital Faisalabad. Int J Sci Eng Res.

[21] Sharif I, Rashid Z, Tariq NA (2019). Gaps in knowledge and practices regarding nosocomial infections among nursing staff of a tertiary care hospital of Rawalpindi. Pak Armed Forces Med J.

[22] Allegranzi B, Storr J, Twyman A (2016). Guidelines on Core Components of Infection Prevention and Control Programmes at the National and Acute Health Care Facility Level. https://apps.who.int/iris/bitstream/handle/10665/251730/9789241549929-eng.pdf.

